# Exploring Ghanaian medical students’ learning experiences during the COVID-19 lockdown: a case study of the University for Development Studies Medical School

**DOI:** 10.12688/f1000research.129653.1

**Published:** 2023-05-12

**Authors:** Anthony Amalba, Bright Yammaha Amoore, Sophia Ewuenye Adwoa Kpebu, Bruce Ayabilla Abugri, Victor Mogre

**Affiliations:** 1 Department of Health Professions Education and Innovative Learning, University for Development Studies, Tamale, Ghana

**Keywords:** Covid-19, health professions education, learning experiences, online learning, lockdown

## Abstract

**Background:** Coronavirus disease (COVID-19) continues to affect health professions education, especially in developing and middle-income countries, even though alternative educational measures have been sanctioned to continue educating students in their homes while observing physical distancing.

**Methods:** A qualitative cross-sectional study design was adopted for the study. Participants among the four departments of a Ghanaian medical school were treated as clusters, and a voluntary response sampling approach was used to recruit students across the clusters to respond to self-administered online Google interview questions on students’ learning experiences during the COVID-19 pandemic.

**Results:** The study found that lack of supervision, lack of access to school library resources, overload of syllabi and the interference of household chores were major factors that made online learning difficult and ineffective during the COVID-19 lockdown. Most participants (n=133, 67%) described online learning as completely inadequate, ineffective and an expensive mode of learning which may not develop the necessary competence and skills required for effective clinical practice.

**Conclusions:** Notwithstanding this, almost all students believed that combining face-to-face and online learning will significantly improve medical education. The COVID-19 pandemic brought about the increased use of online learning in the health professions that was accompanied with significant challenges for students.

## Introduction

Coronavirus disease (COVID-19) is an infectious disease caused by a new coronavirus that has not been previously identified in humans. It was first identified in Wuhan, China on the 23 ^rd^ of December 2019. According to UNICEF, since its discovery, its geographic distribution has continued to evolve. World Health Organization (WHO) reported that globally the outbreak affected 216 countries, with over 13 million cases and 500,000 deaths. Ghana recorded its first two cases from Norway and Turkey returnees on the 12 ^th^ of March 2020, since then the numbers have continued to rise. On the 4 ^th^ of May 2020, Ghana Health Service (GHS) reported 2,719 cases and 18 deaths occurring in 12 out of the 16 regions in the country within two months, amid three weeks of lockdown, intensive screening, and indefinite closure of schools for containment of the virus. Due to the infectious nature of the outbreak many countries attempted to observe the principle of social distancing through resorting to school closure which included health education institutions, to curtail the rising consequences of the outbreak. As shown in UNICEF Ghana report, this affected over 1.2 million learners, representing over 60% of the world’s student population.

COVID-19 continues to affect educational institutions in so many ways even though according to another UNICEF Ghana report alternative educational measures have been adopted through online learning, using various platforms such as zoom, WhatsApp, and Google Classroom in place of the traditional method of instruction (face-to-face delivery) to continue educating students in their homes while observing physical distancing. The approach comes with its challenges, especially in developing countries (low-and middle-income countries) as its ability to raise or maintain the expected quality of health professionals is a concern. For example, in the Philippines, the Commission on Higher Education (CHED) suspended the online form of instruction following resistance from students, including teachers who clamoured against the online mode of learning due to different factors including schools’ lack of preparation for the implementation of these due to inadequate access to internet and online educational resources ( Toquero, 2020).

For a variety of reasons, many schools in the world including Ghana had also suspended all academic activities including clinical clerkships. One reason was to flatten the COVID-19 curve, another reason was to lessen the risk of exposure of student health professionals, although some students may be willing to put themselves at risk to provide support in combat ( Ferrel & Ryan, 2020). According to literature, clinical skills training is a crucial moment in student developmental stage where clinical examination and reasoning skills, including therapeutic communication skills with patients, and multi-disciplinary teams are developed to enhance effective diagnosing and client adherence to treatment ( Adebisi *et al.*, 2020; Cecilio-Fernandes *et al.*, 2020). All of these activities greatly diminished with an online learning environment, which gave no room for real-life clinical practice ( Adebisi *et al.*, 2020; Cecilio-Fernandes *et al.*, 2020). Despite the growing trend of perceived challenges of online learning on the skills development of student health professionals, health educators across the globe continued to stress the need to protect students, arguing that the risks of exposure of students may be greater than the educational benefits of remaining in clinical settings or schools to learn about the new clinical entities, even though their services may be needed in times of healthcare worker shortage ( Anderson *et al.*, 2020). As many student health professionals were missing out on the valuable experiences of presentations, clinical rotations, and collaborative experiences of standards which helped previous generations to become doctors, it poses the question about how these students will evolve and integrate themselves into the medical community given the situation of a prolonged COVID-19 pandemic, or another future incidence of infectious disease ( Ferrel & Ryan, 2020).

Due to the above concerns, there are growing anxieties about the ability of online studies to produce the expected outcome of qualified health professionals, especially in developing and middle-income countries. Though several studies regarding COVID-19 have been conducted so far in relation to the medical field and other fields related to health sciences, very few studies have been undertaken to ascertain the impact of COVID-19 in the field of health professions education in Ghana ( Sintema, 2020). There is thus the need to explore the learning experiences of student health professionals, especially during the adoption of online learning during the COVID-19 lockdown. This study leveraged on the impact of online learning on the professional development of student health professionals to draw recommendations for the overall improvement of the online learning system, with the aim of meeting the standards of health professions education in times of future crisis.

### Research questions

i. How has COVID-19 impacted students’ learning during the COVID-19 lockdown?
ii. How do students perceive the role of online learning in building their competence for future practice?

## Methods

### Study setting

The study was conducted in the University for Development Studies School of Medicine (UDS-SoM), Tamale, Ghana from July-September 2020. It is affiliated to the Tamale Teaching Hospital where students receive clinical training. The UDS-SoM runs four programmes including an MBChB, Doctor of pharmacy, nurse of anaesthesia, and a master’s degree in public health *.* The school uses the problem-based learning (PBL) methodology of teaching for teaching and learning. This approach departs from the traditional teaching format that relies on instructor-formulated lectures with the student being the passive recipient rather than the active researcher of facts. Details of the curriculum is published elsewhere. Like other schools in Ghana and elsewhere, academic activity was brought to a close during the COVID-19 lockdown. There was a break in academic activities for a period as the university was contemplating how to continue with teaching and learning. Subsequently, online teaching tools were rolled out to complete the rest of the academic year.

### Study design, participants, recruitment, and data collection methods

Following a qualitative study approach, participants of the study included all levels of medical students and first- and second-year pharmacy students, given that the pharmacy programme was new and had only two levels of students so far. A voluntary response sampling approach was used to invite participants to participate in the study by sending an electronic link to students’ WhatsApp group platforms. The electronic link contained an introduction to the study, consent procedures and a data collection tool. Those who agreed and consented to the study proceeded to respond to the data collection tool. Students were assured that their participation was voluntary and were at liberty to withdraw from responding to the data collection tool at any point in time. Anonymity and confidentiality were assured by the de-identification of the data collection tool. Data was collected using close-ended and open-ended questionnaires developed using Google forms. The open-ended questions provided students the opportunity to express their opinions and perceptions without restrictions. The open-ended questions were aligned to the research questions and were adapted from those of previous studies. Specifically, students’ perception and opinions on the impact of COVID-19 on their learning and the role of online learning in their professional development as future health care providers. Sex (male, female) which was self-reported by participants, students’ level of training (Level 100 to Level 600) and programme of study were assessed using close-ended questions. Pilot testing of the questions was done among a sample of 10 students to ensure easy comprehensibility. There were no changes made to the study after the pilot study. This study complies with the SRQR guidelines for reporting qualitative research.

The data collection and screening was performed by three of the research team members, BYA, VM and AA. Two team members (BYA and VM) coded the data and generated preliminary themes. The larger research team with expertise in health professions education (VM, AA and BYA), behavioural sciences (SEAK and VM) and qualitative research (SEAK, VM and AA) further deliberated on the suggested codes and themes before approving them as a research team. Differences identified were addressed and adjudicated where necessary.

### Data analysis

Data was exported to Microsoft Excel. All texts were read and re-read by the research team. Coding was independently done by BYA and VM. The codes were then used to generate themes. Codes and themes generated were shared with the other members of the research team for expert advice. The findings were presented according to the themes that were generated.

### Ethical considerations

Ethical approval was obtained from University for Development Studies Institutional Review Board (UDS/IRB/102/20) and participants who gave consent to be part of the study were assured of confidentiality and anonymity.

## Results

A total of 202 students responded to the open-ended questionnaire ( Amalba *et al.,* 2023). Table 1 shows the general characteristics of the students. Participants had a mean age of 23.20 years, and the majority were male. There was no consideration of sex segregation in the design of the study and data analysis because this did not apply to this study, as both males and females had equal access to the online learning system in Ghana.

**Table 1.** General characteristics of the students.

Variable	Frequency (%)
**Sex**	
Male	140(69.3%)
Female	62(30.7%)
**Age**	
17-20	73(36.0%)
21-24	67(33.2%)
25-29	41(20.3%)
30 years and older	21(10.4%)
**Programme**	
MBChB	140(69.3%)
Doctor of Pharmacy	62(30.7%)
**Level of training**	
Level 100	80(39.6)
Level 200	43(21.3)
Level 300	30(14.9)
Level 400	11(5.4)
Level 500	4(2.0)
Level 600	34(16.8)

### Impact of the pandemic on students’ learning in general

Most of the students opined that their learning was made difficult and ineffective during the lockdown because they had no supervision from faculty members, given that they were learning from their homes. Students believed that, for effective learning to be achieved, there is the need for proper supervision and the lack of a supervised environment makes effective learning almost unattainable. This situation made some of them to feel lazy, less motivated and disinterested in learning.

*“I feel lazy to learn or even join for the classes because there's no supervision*”.

“ *It made me reluctant to study since I'm home”.*

Some students opined that, due to the restrictions to movement during the lockdown, their learning was made difficult because they did not have physical access to the University library. According to them the University library has relevant books and other learning resources that are not readily available online.

*“Learning was a little bit challenging especially in accessing the materials. Since it has denied me access to some learning materials due to restricted movement”.*

Students also opined that the swift changes to their usual mode of learning to a situation of learning from their homes was sudden, leaving them confused, struggling and reluctant; this slowed learning at the initial stages. They were, however, quick to add that they managed to adapt to the changes as time went on.

*“It initially slowed my learning process but with time I adapted”.*

*“Everything has moved at a slower pace”.*

Related to the poor supervision, students indicated that learning was made difficult for them during the lockdown since their home environments were full of distractions and interferences, making them unconducive for effective learning. Students opined that their homes were not serene and were rather unfavourable and unsuitable for learning. Some said they were also distracted by house chores.

*“It has not been so great, the pressure at home does not permit effective learning.”*

*“Very bad impact from COVID-19 on my studies. There is a whole lot of home distractions interfering negatively on my studies. Schools should reopen quickly for me to come to school. It has slowed me down…I'm at home and I have a lot of errands to run.”*

*“Well, learning hasn't been easy. With all the chores at home, it has been one long bumpy ride. The home environment is not conducive for learning at this level, considering we (students’) aren't used to it.”*

*“If I'm being honest, this pandemic has truly ruined my style of learning. This is because learning at home is not the same as in school. Schools provide a level of serene environment which is suitable for learning and not in the case of being home.”*

*“COVID-19 has affected my studies negatively. This is because I find it very difficult to study when am at home. The home environment is not very conducive or favourable for learning”.*

Furthermore, students discussed that the lockdown led to a reduction in the amount of time needed to complete the 2019/2020 academic year. According to them, the reduction in the duration meant that contact hours were reduced resulting in overload of syllabi to learn within a short period of time. In addition, they believed some essential syllabi were not covered. All of these, according to the students impeded effective learning.

*“It has affected my studies negatively because we could not complete our curriculum for the 2019/20 academic year. It compressed the normal academic year schedule because of the break we had. It took a certain form of seriousness out. Also, I was forced to learn so much in a short period of time which didn't help”.*

*“It has reduced the effectiveness of my learning **.** It has limited the number of effective contact hours and effective lectures”.*

### Students’ perception of the role of online learning to their training

To make up for the lost instructional periods resulting from the lockdown, new teaching and learning technologies were adopted by both students and lecturers to complete the 2019/2020 academic calendar. Students reported that all their learning was moved online which led to serious challenges for their learning. Students’ opinions regarding the role of online learning on their career training was sought for. Among the 202 students, 198 responded to the question in which only 12% (n=23, 12%) believed online learning will be adequate to help provide them with the required competencies as health professionals. A good number of participants (n=21, 42%) were very sceptical about the ability of online learning to adequately prepare them given the practical-centred nature of their training. And a significant number of them (n=133, 67%) strongly described the online training as completely inadequate, ineffective and an expensive mode of learning even though a considerable number of them also believed an improved electronic learning system will contribute significantly, if not entirely, to the enhancement of the medical education system. Students further opined that the introduction of online learning tools resulted in limited opportunities to acquire practical and clinical skills that required face-to-face meetings with faculty. They recognized that online learning is deficient and cannot develop all the needed competence and skills required for effective clinical practice. Students asserted that, aside the theoretical syllabi, some skills can only be learned by hands on practice, which cannot be entirely acquired by reading and watching videos online. The following quotes further illuminates these views expressed by students.

*“Big No! Sincerely No! Certainly No! I don't feel like a medical student while sitting at home doing online studies. Home is certainly not a medical school. So, I can't be at home to learn medicine. At home where is the medical library? There is none. All the medical books of good quality and standard are in the medical school library. My lecturers are not with me at home. No other place can replace my medical school. I wish to be at school right now”.*

*“Online learning cannot fully complete our training. Aside the theory aspect, medical training requires hands on practice which cannot be acquired online. And even if done online it’s not effective because there is the need for one to practice on something or someone and also see what’s being done well, however, materials for practical and skills are not available at home even if they are there, there is zero supervision”.*

*“I also disagree because, online learning can never replace ward activities where students learn the skill of rapport building for effective communication geared towards diagnosis. Thus, the need for a blend of face-to-face lectures to make such a mark”.*

*“It has negatively affected my learning. This is because, majority of the PBL is based on practical and skills training. All these things were eliminated during the online studies and the core mandate of the PBL, tutorials could not also be effective through this mode of learning, some of the courses are practical oriented and needs much time when emphasizing on it”.*

*“However, having been unable to go to the wards have limited learning especially in a practical sense”.*

*“It has really made it a bit tough because practical sessions are no longer held and I don't get to practice the skills I learn theoretically”.*

*“It has been very bad due to the practical nature of my course. It has slowed down the learning process especially the skills and practical aspects due to zero contacts”.*

### Challenges of online learning during the pandemic

Students indicated that the online learning mode that was rolled out by the University did not work so well for them because they were unable to undertake note revision of their lecture slides, given that they could always search Google for answers online, especially during the end of course assessments.

*“It has affected me negatively in that, it has slowed the level of learning and revision once answers can always be googled”.*

Others described that the process has changed their way of learning.

*“It has changed the whole style of learning for me, I learn better when I go to the ward and observe what I have read being practiced but this was not the case and so it made it more difficult for me to appreciate and understand the topics I read.”*

Students further discussed the absence of physical interactions with lecturers hindered their understanding of concepts since the technological issues provided limited opportunities to ask or answer questions during the teaching and learning process.

*“It has given me another aspect of learning but the Lecturer-student interaction is bad. This is as a result of the methods adopted in order for students to complete the academic year, and some lecturers send in only slides for us to study, it has slowed down learning because there are no lectures to attend for better understanding”.*

Opportunities for social and group interactions were also eliminated. Some students felt that the PBL methodology was not well incorporated in the online teaching and learning, in that, the group discussion that encourages teamwork was eliminated.

*“Badly, not able to meet for group discussion physically. More self-study, as well as group discussions…with minimal practical sessions with supervision”.*

Students identified that online learning came with several cost issues since they will have to spend money to buy data to have internet connectivity. Apart from the cost issues students also opined that there were instances of poor internet connectivity. According to some students, learning became almost impossible with the adaptation of new technologies which was characterized by poor internet connectivity since some of them lived in remote areas of the country.

*“The current electronic learning system is costly and ineffective. This is proved by the challenges we experienced through the online learning process; poor network, expensive cost of data and the environment some of us find ourselves. Sometimes due to bad network, we were unable to hear what is being taught”.*

*“COVID-19 has really slowed my learning pace and also cost me financially. it has been difficult with the online studies due to high cost of data. Oh! Hmm it's not been good at all. E-learning is expensive”.*

*“Negatively, due to bad network and inability to join class. It has been difficult with the online studies due to poor network as it adversely affected my learning with detrimental consequences. It has been difficult, especially for students at the remote area, bad network has been a factor. As a result of the methods adopted in order for students to complete the academic year. Network challenges was a major factor and some lecturers send in only slides for us to study”.*

### Some positive experiences regarding online learning

Apart from the negative impacts of COVID-19 on learning, some students’ felt that there have been some positive experiences they have gained from the adaptation of the new technology in learning leading to the acquisition of new learning experiences.

*“It has broadened my scope on how to learn and communicate online through e-learning.”*

*“It pushed me to explore different avenues to learn, the abrupt end to attending school/classes brought some apathy in studies but it gradually got better with the introduction of the online studies.”*

*“For me, I think the impact is a positive one because it makes us do things in new ways, we thought was not possible in the past.”*

*“It has made most of my learning virtual which requires 90% self-studies.”*

*“It has made my learning very interesting. Adapting to different ways of learning through the online method.”*

*“I would not say that it's been bad. It has been a nice experience with some form of challenges.”*

*“I would say positive, if we consider that this has not been an ordinary time. It has changed the way I learn.”*

*Positive especially with technological way of learning”*

Some students also believed that improved online learning can help to resolve some challenges and make important contributions to medical education. They further believed that the pandemic has impacted their learning positively by enabling them to self-direct their learning, appreciate better the problem-based methodology and focusing more on their books without any interferences as opposed to the usual attendance to lectures, and the hustles of postponement of lectures resulting in a lot of time wasting.

*“At first, I didn't think online learning could be useful for medical education, but now I am convinced if the network and internet access is stable it can positively contribute but not as compared to physical sessions.”*

*“Online learning will ensure that when lecturers or students are indisposed or situations prevent them from coming to class, they can still hold their lectures online; I have realized that some lecturers are sometimes unable to honor their lectures, most of the time, such lessons are either rescheduled or totally cancelled. Since we [doctors] are dealing with human lives, I believe every information from lecturers are needed, so if they can't make it to class physically, they may be able to do it electronically. The online learning will help alleviate classroom acquisition problems in my school, and significantly cut down on the long hours spent in simple classroom lectures. Also, I learn best when I witness things, so through the video lectures submitted by my lecturers, I have been able to appreciate lessons more and I can revise what they say any time.”*

*“It has just made me appreciate the student-centered skills nurtured during preclinical medical school as studies have become self-guided again”*

*“It gave me more time to do self-study than usual times where you have to be going for lectures, tutorials and skills and sometimes lectures will be postponed or no venues resulting in a lot of time wasting. But studying at home, even when lectures are postponed or reschedule, I make proper use of the time than being on campus.”*

*“I am mostly at home due to the COVID-19 protocols, I read more. Helped me focus more on my books.”*

*“Reduced my onsite (bedside and tutorials) learning time increased time for my personal/private studies”*

Several students felt that they were able to learn without pressure and more conveniently on their own by exploring new technologies in learning.

*“I embraced virtual ways of learning, and learning was made comfortable and convenient.”*

*“Disrupted our schedule, but introduced more convenient ways to explore technology for education.”*

*“Honestly, it's better than in school since most of the stressors in school weren't at home. It helped us learn without pressure”*.

## Discussion

In this study we explored the impact of the COVID-19 pandemic/lockdown and its associated online learning on the professional development of student health professionals.

A significant number of the participants (n=133, 67%) strongly described the online learning during the COVID-19 pandemic/lockdown as completely inadequate, ineffective and an expensive mode of learning. Students considered this mode of learning as a sudden, expensive, and challenging change, similar to previous findings reported from the Philippines, where both students and teachers strongly opposed the use of online learning during COVID-19 due to it being an abrupt and expensive initiative that they were not prepared for ( Toquero, 2020). Students incurred extra cost to acquire the needed technological devices and internet services to be able to engage in online learning, which confirms the expensive out-of-pocket expenditure on internet data bundles that medical and pharmacy students in Nigeria also experienced ( Adebisi *et al.*, 2020). This partly contributed to the resistance met by online learning during the COVID-19 pandemic.

Also, due to the practical nature of the training of health professionals, most participants were very sceptical about the ability of online learning to adequately prepare them for future health care practice. For instance, they cited the loss of clinical skills training, laboratory training and the usual clinical rotations in hospitals due to social distancing as major setbacks to their skills development during the COVID-19 pandemic as also shown in a study by Cecilio-Fernandes *et al.*, (2020). Our study further established the findings of previous studies that indicated that online learning poses major challenges to the training of future health professionals ( Adebisi *et al.*, 2020; Ferrel & Ryan, 2020).

Time constraints and the overload of coursework were major effects of COVID-19 lockdown on the learning and professional development of participants in this study. Due to the closure of schools as part of the measure to reduce the spread of the COVID-19 virus, students stayed home for months without any academic work. This delayed the completion of the academic year and placed pressure on students and faculty to do more work in a limited time to catch up on the time lost. This was similar to the experiences of medical and pharmacy students in Nigeria whose studies were also halted, and major professional examinations suspended, due to their government’s measures to curb the spread of the virus ( Adebisi *et al.*, 2020). This could result in low performance in the professional examination post-pandemic and the general competency of these future health professionals and must be given the needed attention.

One challenge also revealed in this study is the lack of supervision and guided learning. The social restrictions during the pandemic took away the face-to-face interactions which included lectures, tutorials, skills training, laboratory training and hospital visitation sessions that students engaged in under the regular PBL module. The shift to online learning took away the guided learning and supervision students usually received from their lecturers in the regular PBL module. It also made it difficult for students to ask questions and get guidance on certain critical topics they found difficult to understand. This made some students feel lazy and less committed to their studies during the lockdown, as found in similar studies where medical students had difficulties in concentrating and learning effectively ( Aftab *et al.*, 2021; Cecilio-Fernandes *et al.*, 2020). This poses a threat to the competencies and readiness of students in their future profession.

Quite similarly, the closure of schools due to COVID-19 and the use of online learning during this period, resulted in less interaction between students and their lecturers as well as their peers. Participants indicated that they missed the regular skills training, laboratory training, hospital visitation and tutorial sessions that allowed them to get practical training and direct interaction with lecturers as well as their peers. As found in other studies, our study revealed that the use of the online lectures and demonstrative videos could not address the needed practical skills students gained from observing and engaging directly with their lecturers ( Cecilio-Fernandes *et al.*, 2020; Ferrel & Ryan, 2020). The collaborative learning at tutorial sessions was also missing and that took away the peer supported learning and social bonding of meeting in groups for tutorials. Thus, the social relations and mental well-being of students which is relevant for future practice was negatively affected ( Adebisi *et al.*, 2020).

The COVID-19 restrictions which led to the closure of schools also limited students’ accessibility to the physical library and other learning resources on campus. Health professionals require certain competencies and skills to be able to work diligently in their profession, therefore their training in school needs to equip them with the best of these competencies and skills. Access to library and other relevant resources complements the lectures and practical training to equip students with the needed knowledge and competencies for their professional practice. This confirms the findings in Nigeria which identified that it was difficult for students to find e-sources for some practical pharmacy and medical courses during the pandemic ( Adebisi *et al.*, 2020). The pandemic therefore challenged the comprehensive professional development of health professional students.

Another worrying situation students faced during the COVID-19 pandemic was the unfavourable learning environment at home. As schools were closed, students had to stay home for some months and resort to online lectures and self-study to cope with their academic work. According to our study, the home did not provide the best environment for effective learning for most students. There were several distractions like chores and other family engagements that did not give them the best study time. Most students also lived in communities that did not have good internet connectivity. Such students were unable to participate effectively in the online lectures the university resorted to. This resonates with in the findings of a similar study in Brazil ( Cecilio-Fernandes *et al.*, 2020). In addition to these challenges, frequent power outages were said to have affected the learning experiences of medical and pharmacy students in Nigeria during the pandemic ( Adebisi *et al.*, 2020). The social restrictions of COVID-19 therefore hindered the conducive learning environment needed for effective learning which further translates into effective professional development for practice.

Amidst reports of the negative impact of COVID-19 on learning among students of health profession at the UDS, a few positive experiences were also identified. Generally, in this study, one positive impact of the online learning system adopted by the institution during the pandemic was the reduction in the person-to-person interactions, which minimized the spread of the virus among the university community as seen in other studies too ( Anderson *et al.*, 2020). This resonates with the findings of other studies that identified the large shift to online instruction as a means of limiting face to face interaction between teachers and the students, to prevent the spread of the virus and preserve lives for the needed future human resources ( Toquero, 2020).

Also, about 12% of the study participants (n=23, 12%) believed that online learning during the COVID-19 pandemic was good. It helped both faculty and students to adapt innovative teaching and learning methods. Just as it was identified by Toquero (2020), this study also revealed that the online learning system adapted during the COVID-19 pandemic caused students to learn and make use of new learning modes to keep up with their learning. Thus, in a positive way, the situation challenged students and faculty to get out of their comfort zone of classroom interaction, to adapt other new technological methods of teaching and learning. Some participants stated that online learning will be adequate to provide them with the required competencies as health professionals if improved electronic learning systems are put in place by the university.

Similarly, the adaption of e-learning during the pandemic also made learning a more student-centred and self-motivated process for students. Our study found that amid the pandemic, students had more time on their hands to engage in self-directed learning. There were no physical classroom interactions which meant that students did not have to face the challenges of travelling to campus as well as missing and rescheduling lectures due to unavailability of lecture rooms or lecturers. This gave students enough time to do more self-studies. Students had more time to study on their own, at their own pace and in the comfort of their homes. This improved personal studies for some students as they were in better control of their studies and were able to explore new learning opportunities to learn new things as also shown in the findings of a similar study in the Philippines ( Toquero, 2020).

## Conclusion

Practical clinical training is an essential part of health professional training. In the wake of technological development and innovative learning, it will be a good thing for higher educational institutions (including health professional training institutions) to identify and utilize innovative ways of teaching practical and clinical skills. This will help maintain the standards and quality of health professional training, even in the wake of other pandemics in future. Educational institutions need to improve technological and internet facilities in their institutions to improve online teaching and learning in this technological era. It is also important for faculty and students to have an effective system of communication in order to give and receive feedback and the needed support with online learning.

## Data Availability

Zenodo: Learning Experiences during COVID-19 lockdown, https://doi.org/10.5281/zenodo.7643568 ( Amalba *et al., *2023) This project contains the following underlying data:
• Learning Experiences during COVID-19 Lockdown.xlsx Data are available under the terms of the Creative Commons Attribution 4.0 International license (CC-BY 4.0).
