# Exploring Ghanaian medical students’ learning experiences during the COVID-19 lockdown: a case study of the University for Development Studies Medical School

**DOI:** 10.12688/f1000research.129653.2

**Published:** 2025-03-17

**Authors:** Anthony Amalba, Bright Yammaha Amoore, Sophia Ewuenye Adwoa Kpebu, Bruce Ayabilla Abugri, Victor Mogre

**Affiliations:** 1 Department of Health Professions Education and Innovative Learning, University for Development Studies, Tamale, Ghana

**Keywords:** Covid-19, health professions education, learning experiences, online learning, lockdown

## Abstract

**Background:**

The COVID-19 pandemic has had a profound impact on health professions education, particularly in developing and middle-income countries. Despite the implementation of alternative educational strategies to facilitate remote learning while maintaining physical distancing, challenges persist in ensuring the effectiveness.

**Methods:**

This qualitative cross-sectional study explored the learning experiences of medical students at a Ghanaian institution during the COVID-19 pandemic. Participants were drawn from four departments within the medical school, using a cluster-based sampling approach. A voluntary response sampling method was employed to recruit students, who completed self-administered online surveys containing interview questions about their educational experiences during the pandemic.

**Results:**

The study identified several barriers to effective online learning, including inadequate supervision, limited access to library resources, overburdened syllabi, and interference from household responsibilities. A significant majority of participants (n=133, 67%) reported that online learning was insufficient, ineffective, and financially burdensome, undermining the development of essential competencies and skills needed for clinical practice.

**Conclusions:**

Notwithstanding these challenges, nearly all participants expressed the belief that a combination of face-to-face and online learning could enhance medical education. The pandemic has accelerated the adoption of online learning in health professions, highlighting the need to address the ongoing challenges faced by students to ensure the effectiveness of this mode of instruction.

## Introduction

Coronavirus disease (COVID-19) is an infectious disease caused by a novel coronavirus that has never been detected in humans. On December 23, 2019, it was discovered in Wuhan, China for the first time. According to UNICEF, its geographical distribution changed rapidly since its discovery. The World Health Organization (WHO) reported that the pandemic touched 216 nations worldwide, with currently over 772 million illnesses and over 6 million fatalities globally ( World Health Organization, 2023). Due to the contagious nature of the outbreak, several nations sought to follow the principle of social distancing by closing schools, including health education institutions, to mitigate the outbreak’s growing implications. On March 12, 2020, Ghana registered its first two cases of returnees from Norway and Turkey, and within a few weeks the number of infections in the country steadily increased. Within two months of the epidemic, the Ghana Health Service (GHS) recorded 2,719 cases and 18 fatalities in 12 of the country’s 16 regions, despite three weeks of lockdown, intense screening, and extended school closures for viral containment. According to the UNICEF Ghana study, this affected over 1.2 million students, accounting for more than 60% of the world’s student population.

During the peak of the COVID-19 pandemic, educational institutions were affected in so many ways even though according to another UNICEF Ghana report alternative educational measures were adopted through online learning, using various platforms such as Zoom, WhatsApp, and Google Classroom in place of the traditional method of instruction (face-to-face delivery) to continue educating students in their homes while observing physical distancing. The approach came with its challenges, especially in developing countries (low-and middle-income countries) as its ability to raise or maintain the expected quality of health professionals was a concern. For example, in the Philippines, the Commission on Higher Education (CHED) suspended the online form of instruction following resistance from students, including teachers who clamored against the online mode of learning due to different factors including schools’ lack of preparation for the implementation of these due to inadequate access to the internet and online educational resources ( Toquero, 2020). According to findings of another study in Pakistan, online learning could not generate the expected outcomes in poor nations like Pakistan, where the vast majority of students are unable to use the internet due to technical and monetary constraints ( Adnan & Anwar, 2020). The results from Adnan and Anwar (2020) also emphasized that the loss of direct engagement with instructors and typical classroom socialization among other challenges hindered effective learning in higher education institutions.

For a variety of reasons, many schools in the world, including Ghana, also suspended all academic activities including clinical clerkships. One of the reasons was to flatten the COVID-19 curve, and another reason was to lessen the risk of exposure to student health professionals, although some students were willing to put themselves at risk to provide combat support ( Ferrel & Ryan, 2020). Research indicates, clinical skills training is a crucial moment in the student developmental stage where clinical examination and reasoning skills, including therapeutic communication skills with patients, and multidisciplinary teams are developed to enhance effective diagnosing and client adherence to treatment ( Adebisi et al., 2020; Cecilio-Fernandes et al., 2020). All of these activities greatly diminished with an online learning environment, which gave no room for real-life clinical practice ( Adebisi et al., 2020; Cecilio-Fernandes et al., 2020). Despite the growing trend of perceived challenges of online learning on the skills development of student health professionals, health educators across the world continued to stress the need to protect students, arguing that the risks of exposure for students may be greater than the educational benefits of remaining in clinical settings or schools to learn about the new clinical entities, even though their services may be needed in times of healthcare worker shortage ( Anderson et al., 2020). As many student health professionals were missing out on the valuable experiences of presentations, clinical rotations, and collaborative experiences of standards which helped previous generations to become doctors, it posed the question of how these students would evolve and integrate themselves into the medical community given the situation of a prolonged COVID-19 pandemic, or another future incidence of infectious disease ( Ferrel & Ryan, 2020). Furthermore, according to a similar research conducted in Ghana, COVID-19 has proven disruptive to traditional medical education in Ghana, though the study concludes that the unique learning processes may offer potential to enhance access to medical education through a phased learning approach ( Asumanu & Tsevi, 2021).

Due to the above concerns about online learning, there were growing anxieties about the ability of online mode of studies to produce the expected outcome of qualified health professionals, especially in developing and middle-income countries. Despite the difficulties the pandemic posed to higher level education and health professional studies, it may be possible to identify opportunities to reevaluate the efficacy of current undergraduate medical education programmes and to embrace novel approaches to delivering instruction while maintaining standards of quality ( Sani et al., 2020). Though several studies regarding COVID-19 have been conducted so far in the medical field and other fields related to health sciences, very few studies have been undertaken to ascertain the impact of COVID-19 in the field of health professions education in Ghana ( Sintema, 2020). There is thus the need to explore the learning experiences of student health professionals, especially during the adoption of online learning during the COVID-19 lockdown. Despite the decline in cases and the continuous vaccination programme being carried out in the country, this study leveraged the impact of online learning on the professional development of student health professionals to draw recommendations for the overall improvement of the online learning system, to meet the standards of health professions education in times of future crisis.

### Research questions

i. How has COVID-19 impacted students’ learning during the COVID-19 lockdown?
ii. How do students perceive the role of online learning in building their competence for future practice?

## Methods

### Study setting

The study was conducted in the University for Development Studies School of Medicine (UDS-SoM), Tamale, Ghana from July-September 2020. It is affiliated to the Tamale Teaching Hospital where students receive clinical training. The UDS-SoM runs four programmes including an MBChB, Doctor of pharmacy, nurse of anaesthesia, and a master’s degree in public health *.* The school uses the problem-based learning (PBL) methodology of teaching for teaching and learning. This approach departs from the traditional teaching format that relies on instructor-formulated lectures with the student being the passive recipient rather than the active researcher of facts. Details of the curriculum is published elsewhere. Like other schools in Ghana and elsewhere, academic activity was brought to a close during the COVID-19 lockdown. There was a break in academic activities for a period as the university was contemplating how to continue with teaching and learning. Subsequently, online teaching tools were rolled out to complete the rest of the academic year.

### Study design, participants, recruitment, and data collection methods

Following a qualitative study approach, participants of the study included all levels of MBChB students and first and second-year pharmacy students, given that the pharmacy program was new and had only two levels of students so far. A voluntary response sampling approach was used to invite participants to participate in the study by sending an electronic link to students’ WhatsApp group platforms ( Murairwa, 2015; Stratton, 2023). The electronic link contained an introduction to the study, consent procedures, and a questionnaire. Those who consented to the study proceeded to respond to the questionnaire. Participants were assured that their participation was voluntary and were at liberty to withdraw from responding to the questionnaire at any point in time. The de-identification of data assured the anonymity and confidentiality of participants. Data was collected using close-ended and open-ended questionnaires developed using Google Forms. The open-ended questions were aligned with the research questions and were adapted from previous studies. Thus, participants gave their perceptions on the impact of COVID-19 on their learning and the role of online learning in their professional development as future healthcare providers. Sex (male, female) which was self-reported by participants, participants’ level of training (Level 100 to Level 600), and program of study were assessed using close-ended questions. Pilot testing of the questions was done among a sample of 10 participants to ensure easy comprehensibility. There were no changes made to the study after the pilot study. This study complies with the SRQR guidelines for reporting qualitative research.

The data collection and screening were performed by three of the research team members, Bright Yammaha Amoore (BYA), Victor Mogre (VM), and Anthony Amalba (AA). Two team members (BYA and VM) coded the data and generated preliminary themes. The larger research team with expertise in health professions education (VM, AA, and BYA), behavioral sciences (Sophia Ewuenye Adwoa Kpebu (SEAK) and VM), and qualitative research (SEAK, VM, and AA) further deliberated on the suggested codes and themes before approving them as a research team. Differences identified were addressed and adjudicated where necessary.

### Data analysis

Data was exported to Microsoft Excel. All texts were read and re-read by the research team. Coding was independently done by BYA and VM. The codes were then used to generate themes. Codes and themes generated were shared with the other members of the research team for expert advice. The findings were presented according to the themes that were generated.

### Ethical considerations

Ethical approval was obtained from University for Development Studies Institutional Review Board (UDS/IRB/102/20) and participants who gave consent to be part of the study were assured of confidentiality and anonymity.

## Results

A total of 202 students responded to the online questionnaire ( Amalba *et al.,* 2023). Table 1 below shows the general demographics of the participants. The mean age of participants was 23.20 years, and the majority were male. Majority of participants were on the MBChB programme and were in the first year (Level 100). There was no consideration of sex segregation in the design of the study and data analysis because this did not apply to this study, as both males and females had equal access to the online learning system in Ghana.

**Table 1.** General characteristics of the students.

Variable	Frequency (%)
**Sex**	
Male	140(69.3%)
Female	62(30.7%)
**Age**	
17-20	73(36.0%)
21-24	67(33.2%)
25-29	41(20.3%)
30 years and older	21(10.4%)
**Programme**	
MBChB	140(69.3%)
Doctor of Pharmacy	62(30.7%)
**Level of training**	
Level 100	80(39.6)
Level 200	43(21.3)
Level 300	30(14.9)
Level 400	11(5.4)
Level 500	4(2.0)
Level 600	34(16.8)

### Perceptions of online learning

To compensate for the missing instructional periods caused by the lockdown, both students and lecturers utilized innovative teaching and learning tools to complete the 2019/2020 academic calendar. Participants noted that all of their learning had been shifted online, posing significant hurdles to their learning. The perspectives of participants on the role of online learning in their career training were solicited and out of 202 participants who consented to participate in the study, 198 participants gave feedback on this question.

Only 23 (12%) of the participants who responded to the question believed online learning would be adequate to help give them with the essential competences as health professionals. Given the practical nature of their training, 42 participants (21%) were hesitant about online learning’s ability to fully prepare them for their future profession. However, they believed an improved electronic learning system would contribute significantly, if not entirely, to the improvement of the medical education system. On the other hand, a significant number of the participants, n=133 (67%) described online training as completely inadequate, ineffective, and costly, in fully preparing them for practice. Participants felt that the advent of online learning tools led in fewer opportunities to gain practical and clinical skills that required face-to-face discussions with faculty. They acknowledged that online learning was inadequate and cannot build all of the necessary competence and skills for effective clinical practice as expressed in the quotes below.

*“It has really made it a bit tough because practical sessions are no longer held and i don’t get to practice the skills I learn theoretically”.* (Participant 62)

*“I also disagree because, online learning can never replace ward activities where students learn the skill of rapport building for effective communication geared towards diagnosis. Thus, the need for a blend of face-to-face lectures to make such a mark”.* (Participant 150)

*“It has been very bad due to the practical nature of my course. It has slowed down the learning process especially the skills and practical aspects due to zero contacts”.* (Participant 161)

### Impact of the pandemic on students’ learning in general

Because students were learning from their homes, the majority of study participants felt that their learning was made difficult and ineffective during the lockdown. Participants believed that sufficient supervision was required for effective learning. They opined that effective learning was almost impossible to do in the absence of a supervised environment. This condition caused some of them to feel lazy, unmotivated, and uninterested in studying, as indicated below.

*“I feel lazy to learn or even join for the classes because there’s no supervision*”. (Participant 51)

“ *It made me reluctant to study since I’m home”.* (Participant 48)

Participants indicated that due to movement limitations during the lockdown, their learning was hampered because they did not have physical access to facilities such as the University library. They said that the University library housed relevant books and other learning tools that were not easily accessible online.

*“Learning was a little bit challenging especially in accessing the materials. Since it has denied me access to some learning materials due to restricted movement”.* (Participant 57)

The participants also opined that the abrupt transition from their typical style of learning to studying from their homes left them confused, uncertain, and reluctant; this delayed learning in the early stages. They did, however, note that they were able to adapt to the changes as time passed.

*“Everything has moved at a slower pace”.* (Participant 119)

*“It initially slowed my learning process but with time I adapted”.* (Participant 114)

Participants added that learning was made difficult for them during the lockdown due to distractions and interferences in their home settings, making them unconducive to good learning. Participants stated that their homes were not serene and were unfit for good learning. Some also opined to be distracted by housework as well.

*“It has not been so great, the pressure at home does not permit effective learning.”* (Participant 27)

*“Well, learning hasn’t been easy. With all the chores at home, it has been one long bumpy ride. The home environment is not conducive for learning at this level, considering we (students’) aren’t used to it.”* (Participant 49)

Furthermore, participants discussed how the lockdown reduced the amount of time needed to complete the 2019/2020 academic year. According to them, the shorter duration meant fewer contact hours, which resulted in an excess of syllabi to learn in a short period of time. Furthermore, they considered that some crucial syllabi were not covered and all of these inhibited effective learning for them.

*“It has affected my studies negatively because we could not complete our curriculum for the 2019/20 academic year. It compressed the normal academic year schedule because of the break we had. It took a certain form of seriousness out. Also, I was forced to learn so much in a short period which didn’t help”.* (Participant 41)

*“It has reduced the effectiveness of my learning. It has limited the number of effective contact hours and effective lectures”.* (Participant 9)

### Challenges of online learning during the pandemic

Participants indicated that the online learning mode that was rolled out by the University did not work so well for them because they were not encouraged to undertake self-study time and revision of their lecture slides, given that they could always search Google for online answers to tests, especially during the end of course assessments. Thus, some participants described that the process changed their way of learning.

*“It has affected me negatively in that, it has slowed the level of learning and revision once answers can always be googled”.* (Participant 82)

*“It has changed the whole style of learning for me, I learn better when I go to the ward and observe what I have read being practiced but this was not the case and so it made it more difficult for me to appreciate and understand the topics I read.”* (Participant 158)

Participants further discussed the absence of physical interactions with lecturers hindered their understanding of concepts since the technological issues provided limited opportunities to ask or answer questions during the teaching and learning sessions online.

*“It has given me another aspect of learning but the lecturer-student interaction is bad. This is as a result of the methods adopted in order for students to complete the academic year, and some lecturers send in only slides for us to study, it has slowed down learning because there are no lectures to attend for better understanding”.* (Participant 44)

Opportunities for social and group interactions were also eliminated. Some participants felt that the PBL methodology was not well incorporated in the online teaching and learning, in that, the group discussion that encourages teamwork and collaborative learning was eliminated.

*“Badly, not able to meet for group discussion physically. More self-study, as well as group discussions…with minimal practical sessions with supervision”.* (Participant 169)

Participants identified that online learning came with several cost issues since they had to spend more money to buy data to have internet connectivity to join online lecture sessions. Apart from the cost issues participants also opined that there were instances of poor internet connectivity. According to some participants, learning became almost impossible with the adaptation of new technologies which was characterized by poor internet connectivity since some of them lived in remote areas of the country.

*“The current electronic learning system is costly and ineffective. This is proved by the challenges we experienced through the online learning process; poor network, expensive cost of data and the environment some of us find ourselves. Sometimes due to bad network, we were unable to hear what is being taught”.* (Participant 30)

*“COVID-19 has really slowed my learning pace and also cost me financially. It has been difficult with the online studies due to high cost of data. Oh! Hmm it’s not been good at all. E-learning is expensive”.* (Participant 67)

### Some positive experiences regarding online learning

Apart from the negative impacts of COVID-19 on learning, some participants felt that there were some positive experiences they gained from the adaptation of the new technology in learning, leading to the acquisition of new learning experiences.

*“It pushed me to explore different avenues to learn, the abrupt end to attending school/classes brought some apathy in studies but it gradually got better with the introduction of the online studies.”* (Participant 100)

*“For me, I think the impact is a positive one because it makes us do things in new ways, we thought was not possible in the past.”* (Participant 77)

Drawing on the positive experiences, some participants also believed that improved online learning could help to resolve some challenges and make important contributions to medical education. They believed that the pandemic had impacted their learning positively by enabling them to self-direct their learning, appreciate better the problem-based methodology and focusing more on their books without any interferences as opposed to the usual attendance to lectures, and the hustles of postponement of lectures resulting in a lot of time wasting.

*“At first, I didn’t think online learning could be useful for medical education, but now I am convinced if the network and internet access is stable it can positively contribute but not as compared to physical sessions.”* (Participant 110)

*“Online learning will ensure that when lecturers or students are indisposed or situations prevent them from coming to class, they can still hold their lectures online; I have realized that some lecturers are sometimes unable to honor their lectures, most of the time, such lessons are either rescheduled or totally cancelled. Since we [doctors] are dealing with human lives, I believe every information from lecturers are needed, so if they can’t make it to class physically, they may be able to do it electronically. The online learning will help alleviate classroom acquisition problems in my school, and significantly cut down on the long hours spent in simple classroom lectures. Also, I learn best when I witness things, so through the video lectures submitted by my lecturers, I have been able to appreciate lessons more and I can revise what they say any time.”* (Participant 18)

Several participants felt that they were able to learn without pressure and more conveniently on their own by exploring new technologies in learning. Thus, it helped them to have better control of their personal study time.

*“I embraced virtual ways of learning, and learning was made comfortable and convenient.”* (Participant 22)

*“Honestly, it’s better than in school since most of the stressors in school weren’t at home. It helped us learn without pressure”* (Participant 193)

The results of the study showed that participants experienced a number of challenges with learning during the lockdown and this resulted in their general uncertainty about online learning in adequately preparing them for their professional practice.

## Discussion

In this study we explored the impact of the COVID-19 pandemic/lockdown and its associated online learning experiences on the professional development of student health professionals.

A significant number of the participants (n=133, 67%) strongly described the online learning during the COVID-19 pandemic/lockdown as completely inadequate, ineffective and an expensive mode of learning. Participants considered this mode of learning as a sudden, expensive, and challenging change, similar to previous findings reported from the Philippines, where both students and teachers strongly opposed the use of online learning during COVID-19 due to it being an abrupt and expensive initiative that they were not prepared for ( Toquero, 2020). Participants in this study incurred extra cost to acquire the needed technological devices and internet services to be able to engage in online learning, which confirms the expensive out-of-pocket expenditure on internet data bundles that medical and pharmacy students in Nigeria also experienced while using online learning during the pandemic ( Adebisi *et al.*, 2020). This partly contributed to the resistance met by online learning during the COVID-19 pandemic.

Also, due to the practical nature of the training of health professionals, most participants were very skeptical about the ability of online learning to adequately prepare them for future health care practice. For instance, they cited the loss of clinical skills training, laboratory training and the usual clinical rotations in hospitals due to social distancing as major setbacks to their skills development during the COVID-19 pandemic, as also shown in a study by Cecilio-Fernandes *et al.* (2020). Our study further established the findings of previous studies that indicated that online learning poses major challenges to the training of future health professionals ( Adebisi *et al.*, 2020; Ferrel & Ryan, 2020).

Time constraints and the overload of coursework were major effects of COVID-19 lockdown on the learning and professional development of participants as reported in this study. Due to the closure of schools as part of the measure to reduce the spread of the COVID-19 virus, students stayed home for months without any academic work. This delayed the completion of the academic year and placed pressure on students and faculty to do more work in a limited time to catch up on the time lost as reported in similar studies elsewhere in Ghana ( Asumanu & Tsevi 2021; Agyei-Nkansah et al., 2020). This was similar to the experiences of medical and pharmacy students in Nigeria whose studies were also halted, and major professional examinations suspended, due to their government’s measures to curb the spread of the virus ( Adebisi *et al.*, 2020). This could result in low performance in the professional examination post-pandemic and the general competency of these future health professionals and must be given the needed attention.

One challenge also revealed in this study is the lack of supervision and guided learning. The social restrictions during the pandemic took away the face-to-face interactions which included lectures, tutorials, skills training, laboratory training and hospital visitation sessions that students engaged in under the regular PBL module. The shift to online learning took away the in-person guided learning and supervision students usually received from their lecturers in the regular PBL module. It also made it difficult for students to ask questions and get guidance on certain critical topics they found difficult to understand. This made some students feel lazy and less committed to their studies during the lockdown, as also found in similar studies where medical students had difficulties in concentrating and learning effectively ( Aftab *et al.*, 2021; Cecilio-Fernandes *et al.*, 2020). This poses a threat to the competencies and readiness of students in their future profession.

Quite similarly, the closure of schools due to COVID-19 and the use of online learning during this period, resulted in less interaction between students and their lecturers as well as their peers. Participants indicated that they missed the regular skills training, laboratory training, hospital visitation and tutorial sessions that allowed them to get practical training and direct interaction with lecturers as well as their peers. As found in other studies, our study revealed that the use of the online lectures and demonstrative videos could not address the needed practical skills students gained from observing and engaging directly with their lecturers ( Cecilio-Fernandes *et al.*, 2020; Ferrel & Ryan, 2020). The collaborative learning at tutorial sessions was also missing and that took away the peer supported learning and social bonding of meeting in groups for tutorials. Thus, the social relations and mental well-being of students which is relevant for future practice was negatively affected ( Adebisi *et al.*, 2020).

The COVID-19 restrictions which led to the closure of schools also limited students’ accessibility to the physical library and other learning resources on campus. Health professionals require certain competencies and skills to be able to work diligently in their profession, therefore their training in school needs to equip them with the best of these competencies and skills. Access to library and other relevant resources complements the lectures and practical training to equip students with the needed knowledge and competencies for their professional practice. This confirms the findings in Nigeria which identified that it was difficult for students to find e-resources for some practical pharmacy and medical courses during the pandemic ( Adebisi *et al.*, 2020). The pandemic therefore challenged the comprehensive professional development of health professional students especially in countries with limited access to advance technology and e-resources.

Another worrying situation students faced during the COVID-19 pandemic was the unfavourable learning environment at home. As schools were closed, students had to stay home for some months and resort to online lectures and self-study to cope with their academic work. According to our study, the home did not provide the best environment for effective learning for most students. There were several distractions like chores and other family engagements that did not give them the best study time. Most students also lived in communities that did not have good internet connectivity. Such students were unable to participate effectively in the online lectures the university resorted to. This resonates with the findings of a similar study in Brazil ( Cecilio-Fernandes *et al.*, 2020). In addition to these challenges, frequent power outages were said to have affected the learning experiences of medical and pharmacy students in Nigeria during the pandemic ( Adebisi *et al.*, 2020). The social restrictions of COVID-19 therefore hindered the conducive learning environment needed for effective learning which further translates into effective professional development for practice.

Amidst reports of the negative impact of COVID-19 on learning among students of health profession at the UDS, a few positive experiences were also identified. Generally, in this study, one positive impact of the online learning system adopted by the institution during the pandemic was the reduction in the person-to-person interactions, which minimized the spread of the virus among the university community as seen in other studies too ( Anderson *et al.*, 2020). This resonates with the findings of other studies that identified the large shift to online instruction as a means of limiting face to face interaction between teachers and the students, to prevent the spread of the virus and preserve lives for the needed future human resources ( Toquero, 2020).

Also, about 12% of the study participants (n=23, 12%) believed that online learning during the COVID-19 pandemic was good. It helped both faculty and students to adapt innovative teaching and learning methods. Just as it was identified by Toquero (2020), this study also revealed that the online learning system adapted during the COVID-19 pandemic caused students to learn and make use of new learning modes to keep up with their learning. Thus, in a positive way, the situation challenged students and faculty to get out of their comfort zone of classroom interaction, to adapt other new technological methods of teaching and learning. Some participants stated that online learning will be adequate to provide them with the required competencies as health professionals if improved electronic learning systems are put in place by the university.

Similarly, the adaption of e-learning during the pandemic also made learning a more student-centred and self-motivated process for students. Our study found that amid the pandemic, students had more time on their hands to engage in self-directed learning. There were no physical classroom interactions which meant that students did not have to face the challenges of travelling to campus as well as missing and rescheduling lectures due to unavailability of lecture rooms or lecturers. This gave students enough time to do more self-studies. Students had more time to study on their own, at their own pace and in the comfort of their homes. This improved personal studies for some students as they were in better control of their studies and were able to explore new learning opportunities to learn new things as also shown in the findings of a similar study in the Philippines ( Toquero, 2020).

## Conclusion

Practical clinical training is an essential part of health professional training. In the wake of technological development and innovative learning, it will be a good thing for higher educational institutions (including health professional training institutions) to identify and utilize innovative ways of teaching practical and clinical skills. This will help maintain the standards and quality of health professional training, even in the wake of other pandemics in future. Educational institutions need to improve technological and internet facilities in their institutions to improve online teaching and learning in this technological era, as blended learning has become more useful even post COVID-19. It is also important for faculty and students to have an effective system of communication in order to give and receive feedback and the needed support with online learning.

## Data Availability

Zenodo: Learning Experiences during COVID-19 lockdown, https://doi.org/10.5281/zenodo.7643568 ( Amalba *et al., *2023) This project contains the following underlying data:
• Learning Experiences during COVID-19 Lockdown.xlsx Data are available under the terms of the Creative Commons Attribution 4.0 International license (CC-BY 4.0).
