# Supplementary material for: Exploring Ghanaian medical students’ learning experiences during the COVID-19 lockdown: a case study of the University for Development Studies Medical School
Source: F1000Res. 2025 Mar 17;12:489. Originally published 2023 May 12. [Version 2] doi: 10.12688/f1000research.129653.2 (PMC12381553; doi:10.12688/f1000research.129653.2)
Supplement: Peer review history

# Reviewer report: Reviewer response for version 2

**DOI:** 10.5256/f1000research.178230.r405891

**Published:** 2025-08-26

**Authors:** Forman Erwin Siagian

**Affiliations:** 1 Department of Medicine, Universitas Kristen Indonesia, East Jakarta, Special Capital Region of Jakarta, Indonesia

In contrast to most fields negatively impacted by COVID, higher education, especially medical education, has actually experienced a blessing in disguise with a shift towards technology-based pedagogy and increased government and institution contributions in the context of strengthening technology and its supporting infrastructure that supports and incorporates e-learning and distance learning [refer to 1. Although the results of this study reveal many barriers faced in implementing remote learning, the unexpressed aspect is the willingness and ability to change old learning patterns to more modern ones which must always open for flexibility [refer to 2] and innovation [refer to 3]. This shows open-mindedness, humility, and limitless honesty in the face of limited conditions. Those noble values ​​in the field of education that should become a universal language for shaping a culture for continuous quality improvement in medical learning [refer to 4], exceeding something that is just merely an analysis of limitations/deficiencies/weaknesses in infrastructure or research methodology or scientific writing.

 Once again, the prestigious journal F1000Research shows respect for the universally accepted values ​​of humanity by accepting this article, even though it was written with many limitations and shortcomings, and how the journal’s values are influential for creating inclusive learning environments and are continously being integrated into medical bulk of science to guide ethical best practice and shape better health outcomes for diverse populations worldwide.

Is the work clearly and accurately presented and does it cite the current literature?

Yes

If applicable, is the statistical analysis and its interpretation appropriate?

I cannot comment. A qualified statistician is required.

Are all the source data underlying the results available to ensure full reproducibility?

Yes

Is the study design appropriate and is the work technically sound?

Yes

Are the conclusions drawn adequately supported by the results?

Yes

Are sufficient details of methods and analysis provided to allow replication by others?

Yes

Reviewer Expertise:

Parasitology. Medical Education

I confirm that I have read this submission and believe that I have an appropriate level of expertise to confirm that it is of an acceptable scientific standard.

## References

[1] Adefuye, A. O., Adeola, H. A., & Busari, J. (2021). The COVID-19 pandemic: the benefits and challenges it presents for medical education in Africa. The Pan African medical journal, 40, 42. https://doi.org/10.11604/pamj.2021.40.42.28489.10.11604/pamj.2021.40.42.28489PMC857191734795823

[2] Barrett, A., Woodward-Kron, R., & Cheshire, L. (2022). Flexibility in primary medical programs: A scoping review. Focus on Health Professional Education, 23(4), 16-34. https://doi.org/10.11157/fohpe.v23i4.579.

[3] Andrews, J. S., & Lomis, K. D. (2021). Expanding innovation from undergraduate to graduate medical education: A path of continuous professional development. Medical Teacher, 43(sup2), S49–S55. https://doi.org/10.1080/0142159X.2021.1935835.10.1080/0142159X.2021.193583534291719

[4] Bendermacher, G. W. G., De Grave, W. S., Wolfhagen, I. H. A. P., Dolmans, D. H. J. M., & Oude Egbrink, M. G. A. (2020). Shaping a Culture for Continuous Quality Improvement in Undergraduate Medical Education. Academic medicine : journal of the Association of American Medical Colleges, 95(12), 1913–1920. https://doi.org/10.1097/ACM.0000000000003406.10.1097/ACM.0000000000003406PMC767866332287081

# Reviewer report: Reviewer response for version 2

**DOI:** 10.5256/f1000research.178230.r405890

**Published:** 2025-08-18

**Authors:** Shahid Rafiq

**Affiliations:** 1 Emerson UniversityMultan, Multan, Pakistan

The main weaknesses of the article lie in the study design, methodological transparency, and alignment between conclusions and findings. Although the study is described as qualitative, it includes substantial quantitative reporting, which creates inconsistency. This should be clarified by presenting the design as exploratory mixed-methods or qualitative descriptive with descriptive statistics, and the rationale for this choice should be explained. The voluntary online sampling approach, limited to students with internet access, introduces self-selection bias that likely underrepresents students in rural areas or with poor connectivity. This limitation should be openly acknowledged and discussed in terms of its impact on transferability.

 Another significant weakness is the limited methodological detail provided about the qualitative analysis. While coding and theme development are described broadly, the absence of a codebook, examples of codes, or measures of inter-coder reliability reduces transparency and hinders reproducibility. To strengthen this, the authors should provide a supplement with the full questionnaire, outline their coding framework, and show how disagreements were resolved. Presenting themes in a clear table with subthemes and representative quotes would also make the analysis more rigorous and easier to follow.

 The literature review is relatively thin and lacks a strong theoretical anchor.

 Although some global and Ghanaian studies are cited, the coverage is not comprehensive, and there is little integration of established frameworks such as the Community of Inquiry or Self-Determination Theory. Expanding the literature base and using a conceptual framework would deepen the analysis and situate the findings within broader discussions of online and blended learning. Additionally, while percentages and frequencies are appropriately used descriptively, the reporting could be strengthened by including denominators, clarifying treatment of missing data, and ensuring that quantitative results are positioned as supportive context rather than definitive evidence.

 Finally, the conclusions extend beyond what the data directly supports. While students clearly perceived online learning as inadequate and preferred blended learning, the claim that blended approaches will “maintain standards and quality” of medical education is overstated, as no competence outcomes were measured. The authors should temper these statements by framing them as student perceptions and recommendations rather than confirmed effects.

Is the work clearly and accurately presented and does it cite the current literature?

Partly

If applicable, is the statistical analysis and its interpretation appropriate?

Partly

Are all the source data underlying the results available to ensure full reproducibility?

Yes

Is the study design appropriate and is the work technically sound?

Partly

Are the conclusions drawn adequately supported by the results?

Partly

Are sufficient details of methods and analysis provided to allow replication by others?

Partly

Reviewer Expertise:

My areas of research are Educational Technology, Higher Education Quality Assurance, and Health Professions Education.

I confirm that I have read this submission and believe that I have an appropriate level of expertise to confirm that it is of an acceptable scientific standard, however I have significant reservations, as outlined above.

# Reviewer report: Reviewer response for version 2

**DOI:** 10.5256/f1000research.178230.r371385

**Published:** 2025-04-07

**Authors:** Linda Tsevi

**Affiliations:** 1 College of Education, University of Ghana, Accra, Greater Accra Region, Ghana

The authors have worked on the comments provided by the reviewer.

Is the work clearly and accurately presented and does it cite the current literature?

Partly

If applicable, is the statistical analysis and its interpretation appropriate?

Partly

Are all the source data underlying the results available to ensure full reproducibility?

Yes

Is the study design appropriate and is the work technically sound?

Partly

Are the conclusions drawn adequately supported by the results?

No

Are sufficient details of methods and analysis provided to allow replication by others?

No

Reviewer Expertise:

Quality assurance in private higher education, online learning.

I confirm that I have read this submission and believe that I have an appropriate level of expertise to confirm that it is of an acceptable scientific standard.

# Reviewer report: Reviewer response for version 1

**DOI:** 10.5256/f1000research.142352.r187318

**Published:** 2023-08-18

**Authors:** Linda Tsevi

**Affiliations:** 1 College of Education, University of Ghana, Accra, Greater Accra Region, Ghana

**Comments to authors:**

 The article examines salient information relating to experiences of students during COVID-19 lockdown in a higher education institution in Ghana. There are some insights and also some problems which are discussed as follows:

1. The clarity of the English language in the paper needs to be improved to enhance readability. There are a number of word repetitions in constructed sentences. It is strongly suggested that the authors use a professional language editor to improve the English and ensure the smooth readability of the article.

**Introduction:**

1. The ‘Introduction’ needs substantial work done on it. For instance, the introduction is written as if the pandemic is still in force. Since the WHO has indicated that COVID-19 is no longer a pandemic, and Ghana, as a country has lifted its COVID-19 restrictions, the introduction should reflect that update. The introduction will be easily readable in the past tense with updated information as most of the happenings indicated, are not being reckoned with in 2023.
2. Data in the ‘introduction’ should be restructured. For example, available statistics indicate that there were more than 500,000 COVID-19 related deaths globally. In the United States of America alone, the deaths were more than 500,000.
3. Page 3. Line 24 - Authors should please change ‘According to literature’ to ‘Research indicates’.
4. Page 3, paragraph 4 – Authors indicate ‘growing anxieties about ability of online studies…’ But the reader is not made aware of some of these ‘growing anxieties.’
5. Authors further indicate that ‘…very few studies have been conducted…’. This assertion is inaccurate as there are a number of related research done, even in the Ghanaian setting. A related paper is titled ‘COVID-19 and Medical Education in Ghana: Assessing the Impact’ by Asumanu E and Tsevi, L. There are additional related studies undertaken. The citations in the ‘introduction’ are scanty.

**Methods:**

1. Parts of the methods are too verbose and require substantial rework.
2. Page 4, Second paragraph, the authors indicate that it is a qualitative study, however the article has substantial quantitative information when one reads further. Authors should indicate if it is a mixed methods study.
3. Authors should explain all acronyms used in the paper and rewrite this section.
4. The Methods section does not have any reference/citation. This an academic paper and not an opinion piece so there is the need for citations to buttress statements made.

**Results:**

1. The themes under the results section read like topics. The themes should give the reader a clear sense of content.  The information in the results section needs to be adequately synthesized and succinct.
2. The way the quotations are interspersed in the paper make their reading very confusing. The reader cannot tell whether the quotation is from one participant or more participants. Authors need to do a lot of rewriting here.
3. Page 5. No reference is made about Table 1 in the paper.
4. This section was not concluded properly. The information ends abruptly.

**Discussion:**

1. There is a vast disconnect in the high writing quality in this section when it is compared to the previous sections where the writing quality is poor. It reads as if this section was written by a different person.

**References:**

1. The references are too scanty in-text. Authors require additional citations on studies done in related areas.

**General Comments:**

1. There is no proper review of literature in this paper so one wonders how the research questions were arrived at.
2. Abstract needs to be rewritten to reflect the revised contents of the paper.
3. This paper requires substantial work before it can be considered for indexing.

Is the work clearly and accurately presented and does it cite the current literature?

Partly

If applicable, is the statistical analysis and its interpretation appropriate?

Partly

Are all the source data underlying the results available to ensure full reproducibility?

Yes

Is the study design appropriate and is the work technically sound?

Partly

Are the conclusions drawn adequately supported by the results?

No

Are sufficient details of methods and analysis provided to allow replication by others?

No

Reviewer Expertise:

Quality assurance in private higher education, online learning.

I confirm that I have read this submission and believe that I have an appropriate level of expertise to state that I do not consider it to be of an acceptable scientific standard, for reasons outlined above.

# Response

**Published:** 2025-02-10

**Authors:** Sophia Ewuenye Adwoa Kpebu

**Affiliations:** University for Development Studies, Ghana

1. The clarity of the English language in the paper needs to be improved to enhance readability. There are a number of word repetitions in constructed sentences. It is strongly suggested that the authors use a professional language editor to improve the English and ensure the smooth readability of the article.

**Response:** The entire paper has been revised and edited by the authors to improve the language and clarity of the study outcomes.

**Introduction:**

 1. The ‘Introduction’ needs substantial work done on it. For instance, the introduction is written as if the pandemic is still in force. Since the WHO has indicated that COVID-19 is no longer a pandemic, and Ghana, as a country has lifted its COVID-19 restrictions, the introduction should reflect that update. The introduction will be easily readable in the past tense with updated information as most of the happenings indicated, are not being reckoned with in 2023.

**Response:** The language of the introduction has been updated with the suggestions given. (Page 1-3)

 2. Data in the ‘introduction’ should be restructured. For example, available statistics indicate that there were more than 500,000 COVID-19 related deaths globally. In the United States of America alone, the deaths were more than 500,000.

**Response**: The data and statistics in the introduction have been updated. (Page 1-3)

 3. Page 3. Line 24 - Authors should please change ‘According to literature’ to ‘Research indicates’.

**Response**: The sentence has been updated as suggested. (Page 3, line 24)

 4. Page 3, paragraph 4 – Authors indicate ‘growing anxieties about ability of online studies…’  But the reader is not made aware of some of these ‘growing anxieties.’

**Response**: The paragraph has been revised. (Page 3, paragraph 4)

 5. Authors further indicate that ‘…very few studies have been conducted…’. This assertion is inaccurate as there are a number of related research done, even in the Ghanaian setting. A related paper is titled ‘COVID-19 and Medical Education in Ghana: Assessing the Impact’ by Asumanu E and Tsevi, L. There are additional related studies undertaken. The citations in the ‘introduction’ are scanty.

**Response:** The literature review forms part of the introduction  and it has been updated with references from related that were available at the time the paper was written and submitted. (page 1-3)

**Methods:**

 1. Parts of the methods are too verbose and require substantial rework.

**Response:** The methods section has been reviewed as suggested. (Page 4)

 2. Page 4, Second paragraph, the authors indicate that it is a qualitative study, however the article has substantial quantitative information when one reads further. Authors should indicate if it is a mixed methods study.

**Response:** The information on the study type has been updated. (Page 4, second paragraph.)

 3. Authors should explain all acronyms used in the paper and rewrite this section.

**Response:** All acronyms used in the manuscript have been duly explained.

 4. The Methods section does not have any reference/citation. This an academic paper and not an opinion piece so there is the need for citations to buttress statements made.

**Response**: The methods section has been updated with relevant references. (Page 4)

**Results:**

 1. The themes under the results section read like topics. The themes should give the reader a clear sense of content.  The information in the results section needs to be adequately synthesized and succinct.

**Response**: The results section has been reviewed as suggested.

 2. The way the quotations are interspersed in the paper make their reading very confusing.

 The reader cannot tell whether the quotation is from one participant or more participants. Authors need to do a lot of rewriting here.

**Response**: The quotations for the results section have been duly revised with participant IDs included.

 3. Page 5. No reference is made about Table 1 in the paper.

**Response:** The reference for Table 1 has been updated.

 4. This section was not concluded properly. The information ends abruptly.

**Response**: A conclusion for results section has been provided. (Page 8)

**Discussion:**

 1. There is a vast disconnect in the high writing quality in this section when it is compared to the previous sections where the writing quality is poor. It reads as if this section was written by a different person.

**Response**: The discussion section has been reviewed as suggested, reflecting the main outcomes of the study.

**References:**

 1. The references are too scanty in-text. Authors require additional citations on studies done in related areas.

**Response:** The citations and referencing of other studies related to this paper at the time of submission have been provided. The section has been been duly updated.

**General comments:**

 1. There is no proper review of literature in this paper so one wonders how the research questions were arrived at.

**Response:** The introduction section of the paper provides a review of related literature, based on which the research questions were developed.

 2. Abstract needs to be rewritten to reflect the revised contents of the paper.

**Response: **The abstract has been duly revised to reflect the content of the paper

 3. This paper requires substantial work before it can be considered for indexing.

**Response: **The entire paper has been revised, incorporating the suggestions from the reviewer.
